# British Medical Association—Papers Read, Etc.

**Published:** 1871-10

**Authors:** 


THE following abstracts of some of the papers read at the meeting of the British Medical Association at Plymouth, on the 8th, 9th, 10th, and 11th of August, are taken from *The Doctor:*

"Comparative Advantages of Laryngoscopic Treatment, and Direct Incision into the Larynx," formed the subject of a paper by Dr. Morell Mackenzie. He stated that the relative advantages of these two methods must be considered in relation (1) to the quickness of cure, (2) completeness of removal and probability of recurrence, (3) danger to life, and (4) restoration of voice. From an experience of 100 cases treated, a month was estimated to be the average duration of laryngoscopic treatment. External treatment, on the other hand, required only a fortnight. As regards the second question, complete removal was able to be effected in 97 per cent of the cases which underwent the full course of laryngoscopic treatment, and recurrence took place in about 7 per cent. In twenty-eight cases of direct incision, collected from all sources, ten died in a short time) and in the remaining eighteen the growth was incompletely removed in three cases, and recurrence took place in three cases, or, in other words, in 20 per cent. As regards danger in life, no death occurred in the laryngoscopic cases, whereas, of the twenty-eight treated by external operation, three immediately terminated fatally, six died at the end of a few months, and one from an independent disease. With reference to restoration of function, perfect voice was regained in 77 per cent. of those who underwent laryngoscopic treatment, and a more or less serviceable voice was restored in 16 per cent. Of the eighteen patients who survived direct incision more than a few months, only nine* completely recovered their voice, four had persistent hoarseness, and four permanent aphonia. Consideration of the above statistics establishes the permanent value of laryngoscopic methods of treatment, and justifies one in saying that extra-laryngeal treatment ought never to be adopted unless there be danger to life from suffocation or disphagia.

Dr. R. W. Crichton read a paper on "The Value of the Sulphate of Iron as a Local Application in Phlegmasia Dolens." This method of treatment was first adopted by the author many years ago, from the great success reported by Velpeau from its use locally in erysipelas. It had been employed exclusively in that form of phlegmasia dolens commencing at the calf of the leg and extending upwards to the groin, where the veins are chiefly involved. It had been applied as a lotion (twenty or thirty grains to one ounce of water) as hot as the patient could comfortably bear it, generally by means of spongio-piline. All the cases so treated had made good and rapid recoveries, contrasting favorably with cases formerly treated by leeching and hot fomentations. Muriated tincture of iron was, at the same time, given in large doses. The same method of treatment was suggested in other cases of phlebitis.

"The Lesions of Enteric Fever as an Occasional Cause of Permanent Injury to Nutrition" was spoken of by Dr. Clifford Allibutt. He drew attention to the convalescence from enteric fever, which is well known to be often so tedious; and he raised the question whether the specific lesions of that disease, affecting as they do the instruments of absorption, might not sometime be the cause of permanent marasmus. In enteric fever the local mischief falls not only upon the patches of Peyer in the ileum, but spreads itself throughout the network of the mesentery. If a rat be fed upon tallow candles and then killed, the presence of fat in great quantities in the mesenteric network and glands shows how active is that system in taking up this element of nutrition. Any disease, therefore, within it, or chronic peritonitis outside it, would have its visible effect in

*These cases are tabulated in the Thyrotomy table in the author's "Essay on Growths in the Larynx." In this table, however, the result of case 27 is entered as 'not stated,' but since the publication of the volume of Dr. S. Cohen has informed the author that the result "was complete restoration of voice."

hindering the absorption of fat and in preventing the laying on of adipose tissue. These considerations occurred to the author in consequence of his advice being sought in several cases of marasmus, pure and simple, without local disease, without fever, and without adequate loss of appetite. In all of those a severe attack of enteric fever had preceded the marasmus. The patients, who were almost denuded of adipose tissue, had, previous to the attack of fever been in good health.

